# Retention in HIV Care between Testing and Treatment in Sub-Saharan Africa: A Systematic Review

**DOI:** 10.1371/journal.pmed.1001056

**Published:** 2011-07-19

**Authors:** Sydney Rosen, Matthew P. Fox

**Affiliations:** 1 Center for Global Health and Development, Boston University, Boston, Massachusetts, United States of America; 2 Health Economics and Epidemiology Research Office, Wits Health Consortium, Faculty of Health Sciences, University of the Witwatersrand, Johannesburg, South Africa; 3 Department of Epidemiology, Boston University School of Public Health, Boston, Massachusetts, United States of America; Duke University Medical Center, United States of America

## Abstract

**Background:**

Improving the outcomes of HIV/AIDS treatment programs in resource-limited settings requires successful linkage of patients testing positive for HIV to pre–antiretroviral therapy (ART) care and retention in pre-ART care until ART initiation. We conducted a systematic review of pre-ART retention in care in Africa.

**Methods and Findings:**

We searched PubMed, ISI Web of Knowledge, conference abstracts, and reference lists for reports on the proportion of adult patients retained between any two points between testing positive for HIV and initiating ART in sub-Saharan African HIV/AIDS care programs. Results were categorized as Stage 1 (from HIV testing to receipt of CD4 count results or clinical staging), Stage 2 (from staging to ART eligibility), or Stage 3 (from ART eligibility to ART initiation). Medians (ranges) were reported for the proportions of patients retained in each stage. We identified 28 eligible studies. The median proportion retained in Stage 1 was 59% (35%–88%); Stage 2, 46% (31%–95%); and Stage 3, 68% (14%–84%). Most studies reported on only one stage; none followed a cohort of patients through all three stages. Enrollment criteria, terminology, end points, follow-up, and outcomes varied widely and were often poorly defined, making aggregation of results difficult. Synthesis of findings from multiple studies suggests that fewer than one-third of patients testing positive for HIV and not yet eligible for ART when diagnosed are retained continuously in care, though this estimate should be regarded with caution because of review limitations.

**Conclusions:**

Studies of retention in pre-ART care report substantial loss of patients at every step, starting with patients who do not return for their initial CD4 count results and ending with those who do not initiate ART despite eligibility. Better health information systems that allow patients to be tracked between service delivery points are needed to properly evaluate pre-ART loss to care, and researchers should attempt to standardize the terminology, definitions, and time periods reported.

*Please see later in the article for the Editors' Summary*

## Abstract 2

In this systematic review, Sydney Rosen and Matthew Fox find that less than one-third of patients who tested positive for HIV, but were not eligible for antiretroviral therapy (ART) when diagnosed, were retained in pre-ART care continuously.

## Editors' Summary

**Background:**

Since 1981, AIDS has killed more than 25 million people, and about 33 million people (mostly living in low- and middle-income countries) are now infected with HIV, the virus that causes AIDS. HIV gradually destroys immune system cells (including CD4 cells, a type of lymphocyte), leaving infected individuals susceptible to other infections. Early in the AIDS epidemic, most HIV-infected people died within ten years of infection. Then, in 1996, highly active antiretroviral therapy (ART) became available, and, for people living in developed countries, HIV infection became a chronic condition. Unfortunately, ART was extremely expensive, and HIV/AIDS remained a fatal illness for people living in developing countries. In 2003, governments, international agencies, and funding bodies began to implement plans to increase ART coverage in resource-limited countries. By the end of 2009, about a third of the people in these countries who needed ART (HIV-positive people whose CD4 count had dropped so low that they could not fight other infections) were receiving treatment.

**Why Was This Study Done?:**

Unfortunately, many HIV-positive people in resource-limited countries who receive ART still do not have a normal life expectancy, often because they start ART when they have a very low CD4 count. ART is more successful if it is started before the CD4 count falls far below 350 cells/mm^3^ of blood, the threshold recommended by the World Health Organization for ART initiation. Thus, if the outcomes of HIV/AIDS programs in resource-limited settings are to be improved, all individuals testing positive for HIV must receive continuous pre-ART care that includes regular CD4 counts to ensure that ART is initiated as soon as they become eligible for treatment. Before interventions can be developed to achieve this aim, it is necessary to understand where and when patients are lost to pre-ART care. In this systematic review (a study that uses predefined criteria to identify all the research on a given topic), the researchers investigate the retention of HIV-positive adults in pre-ART care in sub-Saharan Africa.

**What Did the Researchers Do and Find?:**

The researchers identified 28 studies that included data on the proportion of adult patients retained between any two time points between testing positive for HIV and starting ART in HIV/AIDS care programs in sub-Saharan Africa. They defined three stages of pre-ART care: Stage 1, the interval between testing positive for HIV and receiving CD4 count results or being clinically assessed; Stage 2, the interval between enrollment in pre-ART care and the determination of eligibility for ART; and Stage 3, the interval between being deemed eligible for ART and treatment initiation. A median of 59% of patients were retained in Stage 1 of pre-ART care, 46% were retained in Stage 2, and 68% were retained in Stage 3. Retention rates in each stage differed greatly between studies—between 14% and 84% for Stage 3 pre-ART care, for example. Because the enrollment criteria and other characteristics of the identified studies varied widely and were often poorly defined, it was hard to combine study results. Nevertheless, the researchers estimate that, taking all the studies together, less than one-third of patients testing positive for HIV but not eligible for ART when diagnosed were retained in pre-ART care continuously.

**What Do These Findings Mean?:**

These findings suggest that there is a substantial loss of HIV-positive patients at every stage of pre-ART care in sub-Saharan Africa. Thus, some patients receiving a positive HIV test never return for the results of their initial CD4 count, some disappear between having an initial CD4 count and becoming eligible for ART, and others fail to initiate ART after having been found eligible for treatment. Because only a few studies were identified (half of which were undertaken in South Africa) and because the quality and design of some of these studies were suboptimal, the findings of this systematic review must be treated with caution. In particular, the estimate of the overall loss of patients during pre-ART care is likely to be imprecise. The researchers call, therefore, for the implementation of better health information systems that would allow patients to be tracked between service delivery points as a way to improve the evaluation and understanding of the loss of HIV-positive patients to pre-ART care in resource-limited countries.

**Additional Information:**

Please access these Web sites via the online version of this summary at http://dx.doi.org/10.1371/journal.pmed.1001056.

- Information is available from the US National Institute of Allergy and Infectious Diseases on HIV infection and AIDS
- HIV InSite has comprehensive information on all aspects of HIV/AIDS
- Information is available from Avert, an international AIDS charity on many aspects of HIV/AIDS, including information on HIV/AIDS treatment and care, on HIV and AIDS in Africa and on universal access to AIDS treatment (in English and Spanish)
- The World Health Organization provides information about universal access to AIDS treatment, including the 2010 progress report (in English, French and Spanish); its 2010 ART guidelines can be downloaded (in several languages)
- The International AIDS Economics Network posts information about economic, social, and behavioral aspects of HIV care and treatment
- Up-to-date research findings about HIV care and treatment are summarized by NAM/aidsmap

## Introduction

The remarkable expansion of access to antiretroviral therapy (ART) for HIV/AIDS in resource-constrained countries has given nearly four million HIV-positive adults in sub-Saharan Africa the opportunity to achieve what for many may be nearly normal life expectancies [1]. Others, however, do not make it past their first year on treatment. The rate of early mortality and loss to follow-up, which itself portends mortality for many, averages 23% across the region [2]. For patients initiating ART late, with very low CD4 counts, the odds of success are even lower: in a pooled analysis of data from multiple resource-limited countries, patients with starting CD4 counts below 25 cells/mm^3^ faced a more than 3-fold increased risk of death compared to those with starting CD4 counts above 50 cells/mm^3^ [3]. Those who survive suffer more morbidity and utilize more medical care resources than would otherwise have been necessary [4].

Earlier initiation of ART requires earlier diagnosis and regular monitoring until treatment eligibility. Despite large-scale HIV testing campaigns to hasten diagnosis [5] and the raising of CD4 count thresholds to allow earlier ART eligibility [1], late presentation for AIDS treatment remains the norm. Median baseline CD4 counts have increased only modestly in the years since treatment became available [6],[7], and most programs still report medians well below even the very low threshold of 200 cells/mm^3^ previously allowed by most treatment guidelines [2].

The persistence of low starting CD4 counts points to a problem that has just begun to be recognized in the research literature: poor pre-ART retention in care, or the failure to link patients from HIV testing to HIV care and retain them in care until they are eligible for ART. Without effective retention in pre-ART care, beginning with HIV testing and continuing until the first antiretrovirals are dispensed, even patients who have long been aware of their HIV status will access care only when seriously ill, which is often well after treatment eligibility.

A prerequisite to developing interventions to retain patients in care between testing and treatment is an understanding of where and when they are being lost. Research on retention in pre-ART care is challenging, as it requires long periods of follow-up and consistent information systems that allow individuals to be tracked as they move in and out of care at multiple facilities. As a result, only a handful of quantitative studies reporting on rates of pre-ART linkage and loss have been published. In this paper, we review those studies and summarize what is known about this issue in sub-Saharan Africa. Our objective is to determine whether existing data allow us to estimate what proportion of adult patients who test positive for HIV are staged, enroll, and remain in pre-ART care until ART-eligible, and initiate ART as soon as eligible.

## Methods

### Ethics Statement

An ethics statement was not required for this work.

### Search Strategy

We conducted a systematic literature review of patient retention between HIV testing and ART initiation in sub-Saharan Africa. Following a detailed search protocol and standard systematic review procedures (Texts S1 and S2), we searched the published literature and major conference abstract archives for reports containing primary, patient- or facility-level data from routine health-care delivery settings on the proportion of patients retained in care between HIV testing and ART initiation and/or rates of linkage between any two intermediate points between testing and ART. We excluded patients who were in care solely for the purpose of preventing mother-to-child transmission of HIV, patients who were in pediatric care, modeled estimates without primary data, qualitative studies, and clinical trials that did not take place under routine care conditions. We included reports of trials of procedural changes within facilities. Where multiple reports described the same data, the one reporting the most complete follow-up or with the clearest definitions of outcomes was used. We did not place a language restriction on the papers included in our search but did limit the search to English-language indices.

We searched PubMed and the ISI Web of Knowledge through January 5, 2011, with the combined terms “Africa” and “HIV” plus “retention,” “linkage,” or “pre-ART.” We searched the African Indicus Medicus through April 1, 2011, using the same terms. We also searched abstracts from the conferences of the International AIDS Society from 2008 to 2010 and from the Conference on Retroviruses and Opportunistic Infections from 1997 to 2011, and scanned the titles of abstracts presented at the HIV Implementers Meetings in 2008 and 2009 and the 5th International Conference on HIV Treatment Adherence (2010). Finally, we reviewed the reference lists of all papers found through the PubMed and ISI Web of Knowledge searches.

S. R. assessed the eligibility of all abstracts and journal articles that met our initial criteria, and M. P. F. confirmed eligibility. Using a standard data extraction form, both authors extracted and reviewed the relevant data, including study site, sample size and inclusion criteria, dates of data collection, study design and outcomes, and quantitative results.

### Data Analysis

We anticipated that wide variation in definitions, outcomes, and specific components of pre-ART care evaluated in the studies would prevent aggregate statistical analysis of findings beyond a basic descriptive level. We therefore began by describing each study, identifying the start and end points of the data presented, and specifying the proportions of patients retained or linked. We defined “loss to care” as failing to reach the next step in the care sequence for any reason (death or discontinuation), but we also accepted each study's own criteria for determining which patients died or discontinued care. Transfers were rarely distinguished from losses in the published studies. Where possible, we used the reported data to calculate a 95% confidence interval for the proportion of patients retained or linked. Next, we grouped the findings into stages within the testing-to-ART-initiation sequence, as described below, and illustrated the results using forest plots. Finally, for each stage we estimated the median proportion of patients completing the stage and reported the median and range.

### Classification of Results

**Figure 1:**
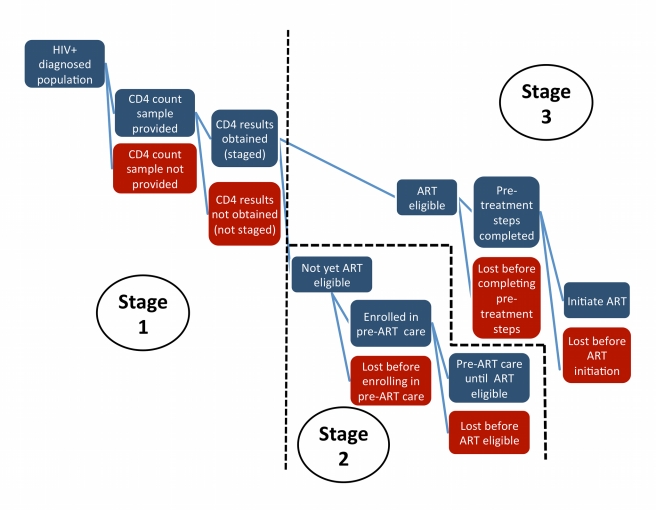
Stages of pre-ART care.

Preliminary review of the literature suggested that the sequence of events that starts with testing positive for HIV and ends with initiating ART can usefully be grouped into three stages, as illustrated in Figure 1. For analysis, we categorized each study by stage, allowing some studies to be included in more than one stage as appropriate.

Stage 1, in which the patient is staged for referral to either pre-ART care or ART, starts immediately after a patient tests positive for HIV infection. Depending on the technology available and the testing setting, Stage 1 typically requires the patient to make one or two additional visits to a clinic. A blood sample for a CD4 count can be given during the same visit as the HIV test if the test is conducted at a clinic; if the test is done at a stand-alone testing site, the patient is typically referred elsewhere to provide a blood sample. Once the sample has been taken, patients are asked to return 2 d to 2 wk later to receive their results, with the time interval dependent on laboratory processing capacity and location. Completion of Stage 1 requires that patients receive their CD4 count results (or clinical staging outcome) and be referred onward for pre-ART care or ART.

Stage 2 lasts from enrollment in pre-ART care until eligibility for ART. Stage 2 pertains only to patients who complete Stage 1 prior to ART eligibility, as those already eligible for ART at staging will be referred directly to Stage 3. The steps included in Stage 2 are generally poorly defined in the literature and vary widely from program to program. In some programs “enrollment in care” happens automatically when a patient presents at a site, regardless of patient intention, while in others it requires active patient participation. Patients may be considered enrolled in care prior to staging or only after having been found not-yet-eligible for ART. At a minimum, retention in pre-ART care requires regular clinic visits for monitoring of patient condition. The frequency and content of these visits varies widely: patients with very high CD4 counts may be asked to return as infrequently as once a year, while those approaching treatment eligibility may be monitored on a monthly or quarterly basis. Similarly, some programs routinely dispense cotrimoxazole, isoniazid, vitamins, and/or food supplements to pre-ART patients, while others simply assess condition. For practical purposes, completion of Stage 2 requires that ART eligibility be determined prior to the patient's CD4 count falling substantially below the eligibility threshold or the patient becoming severely ill.

Finally, Stage 3 encompasses the steps between determination of ART eligibility and ART initiation. Programs in sub-Saharan Africa typically require two or more “treatment readiness” visits during this stage, and the full course of treatment education and adherence training can last for up to 8 wk. Completion of Stage 3 requires that the patient be dispensed a first dose of antiretrovirals.

## Results

**Figure 2:**
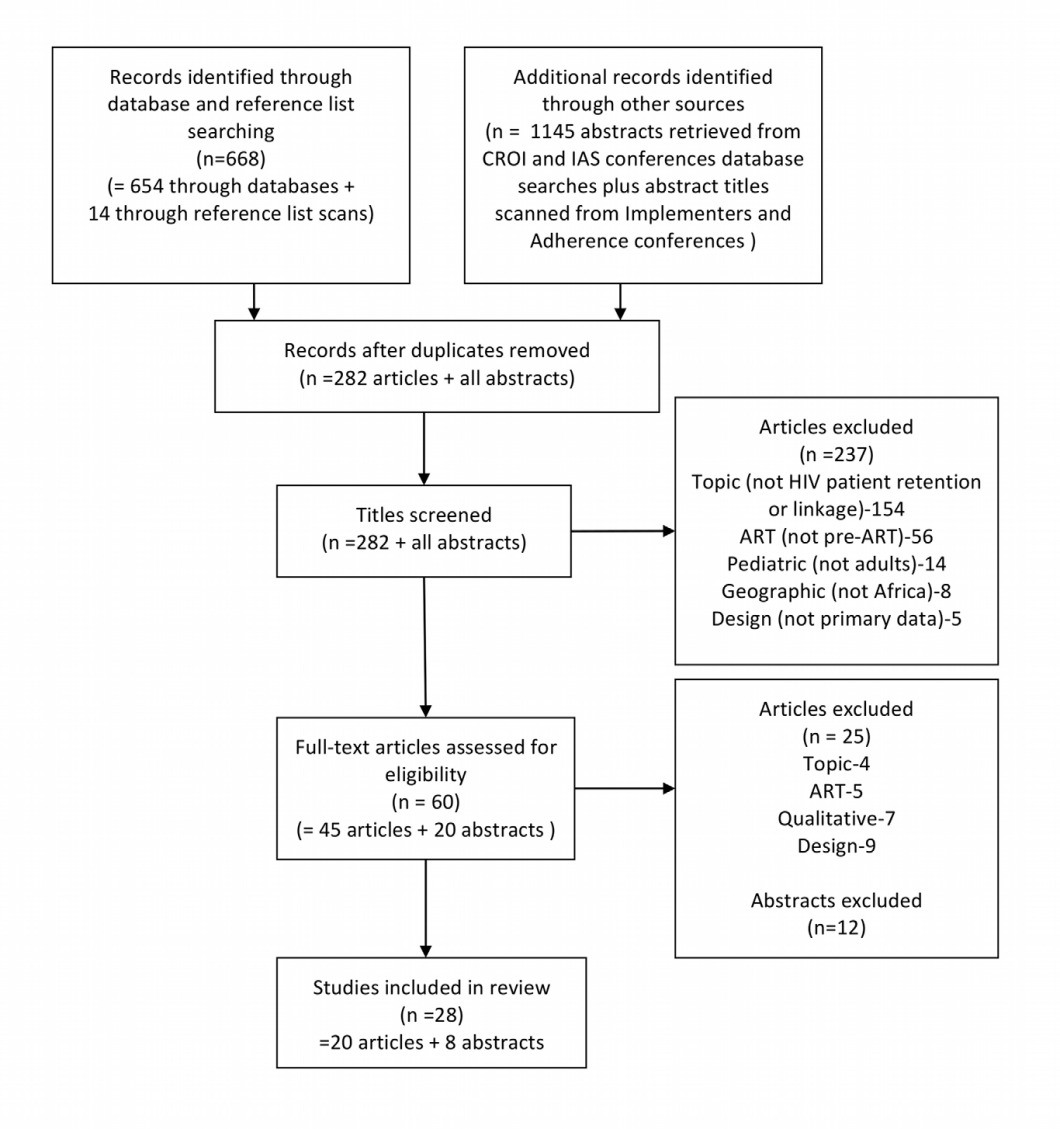
Flow chart of literature search on pre-ART retention in care. Adherence conference, 5th International Conference on HIV Treatment Adherence; CROI, Conference on Retroviruses and Opportunistic Infections; IAS, International AIDS Society; Implementers conference, HIV Implementers Meetings.

We identified 668 full-length journal articles and 1,145 abstracts potentially relevant to our review. As shown in the search flowchart in Figure 2, after excluding duplicates and studies that did not meet the geographic, population, content, or design criteria of our review, 20 full-length articles and eight abstracts were eligible for the review. Most (23/28) were published or presented in 2009 or later. Seven countries are represented, but half the studies (14/28) were conducted in just one, South Africa. Most (18/28) were designed as retrospective cohorts using routinely collected patient-level data; the remaining were program evaluations, trials of procedural changes, and a prospective cohort. The studies are described in Table 1, which also contains the study codes we will use to refer to individual studies throughout this paper. Of the 28 studies included, 20 reported information relevant to only one stage in the testing-to-treatment sequence, six addressed two stages, and two addressed to all three stages. We thus had a total of 38 stage-specific observations.

**Table 1:** Studies included in this review of retention in pre-ART HIV care in sub-Saharan Africa.

Study Code	Year	Location	Sample (*N*)	Dates	Design
Ethiopia 1 [16]	2010	Ethiopia: national sample of public sector sites	HIV+ patients referred for care (1,314)	2005–2008	Evaluation of aggregate site-level reports
Ethiopia 2 [17]	2009	Ethiopia: 33 public sector facilities	HIV+ patients referred for care (1,102)	Jan–Dec 2008	Evaluation of improved referral procedures through collection of referral slips brought to referral clinic by patients after testing
Ethiopia 3 [18]	2010	Ethiopia: Arba Minch Hospital	HIV+ patients presenting at HIV clinic (2,191)	Jan 2003–31 Dec 2008	Retrospective cohort
Kenya 1 [19]	2007	Kenya: Migori District Hospital, Nyanza Province	ART-eligible patients from PMTCT program (159)	Apr 2004–Sep 2005	Retrospective cohort; limited to PMTCT participants and partners
Kenya 2 [20]	2011	Kenya: multiple facilities, Nyanza Province	HIV+ patients accepting home-based testing and follow-up interview (737)	Feb 2008–Jul 2009	Household survey of participants in home-based HIV testing study; self-reported data
Kenya 3 [14]	2011	Kenya: Coptic Hope Center for Infectious Diseases, Nairobi	ART-ineligible patients enrolled in pre-ART care program with a baseline CD4 count (610)	2005–2007	Retrospective cohort
Malawi 1 [21]	2010	Malawi: Martin Preuss Centre, Bwaila District Hospital, Lilongwe	ART-eligible pregnant women referred from PMTCT site to ART site (742)	Dec 2006–Jan 2010	Retrospective cohort
Malawi 2 [22]	2010	Malawi: Thyolo District Hospital	All newly registered care patients in WHO stages 1/2 not on ART and enrolled >1 mo before data censoring (1,428)	1 Jun 2008–10 Feb 2009	Retrospective cohort
Malawi 3 [23]	2006	Malawi: Thyolo District Hospital	HIV+ TB patients who completed first 8 wk of TB treatment and became eligible for ART (742)	Feb 2003–Jul 2004	Retrospective cohort; limited to TB patients
Mozambique 1 [24]	2009	Mozambique: two urban HIV care networks	HIV+ patients (6,999)	1 Jul 2004–30 Jun 2005	Facility-level analysis of numbers completing each step
SA 1 [25]	2009	South Africa: two clinics, Cape Town township	HIV+ patients (375); ART-eligible patients (75)	2006^a^	Retrospective cohort; excluded pregnant women
SA 2 [26] b	2009	South Africa: McCord Hospital, Durban	ART-eligible adults who stated intention to start ART at site and were assessed as “psychosocially ready” for treatment (501)	Jul–Dec 2006^c^	Retrospective cohort
SA 3 [27] b	2010	South Africa: McCord and St. Mary's Hospitals, Durban	HIV+ patients (1,474)	Nov 2006–Jun 2009	Prospective cohort
SA 4 [28]	2010	South Africa: 36 facilities, Free State Province	Patients enrolled in care with CD4 count reported (33,122)	May 2004–Dec 2008	Retrospective cohort
SA 5 [29]	2008	South Africa: Hannan Crusaid Treatment Centre, Gugulethu	ART-eligible patients (2,131)	1 Sep 2002–30 Sep 2007	Retrospective cohort; limited to female patients
SA 6 [30]	2010	South Africa: Cape Town township public clinic	HIV+ patients (988)	Jan 2004–Mar 2009	Retrospective cohort
SA 7 [31]	2010	South Africa: Themba Lethu Clinic, Helen Joseph Hospital, Johannesburg	HIV+ patients (416)	Jan 2008–Feb 2009	Retrospective cohort
SA 8 [32]	2010	South Africa: Themba Lethu Clinic, Helen Joseph Hospital, Johannesburg	Patients enrolled in pre-ART care program (356)	Jan 2007–Feb 2008	Retrospective cohort
SA 9 [33]	2006	South Africa: Gugulethu Community Health Centre, Western Cape Province	ART-eligible patients enrolled at ART clinic (1,235)	Sep 2002–Aug 2005	Retrospective cohort
SA 10 [34]	2010	South Africa: Hlabisa Care and Treatment Program, KwaZulu Natal Province	HIV+ patients not eligible for ART (4,223)	1 Jan 2007–30 Jan 2009	Retrospective cohort
SA 11 [35] b	2010	South Africa: McCord and St. Mary's hospitals, Durban	HIV+ patients (454)	Nov 2006–May 2007	Prospective cohort
SA 12 [36]	2010	South Africa: Gauteng Province	HIV+ patients who enrolled in trial (199)	Not reported	Preliminary data for cohort enrolled in trial; self-reported data; limited to female IDUs and CSWs
SA 13 [37]	2010	South Africa: Esselen St. Clinic, Hillbrow, Johannesburg	HIV+ patients (224)	Not reported	Trial of immediate or 1-wk CD4 results; source reported only on 1-wk outcomes
SA 14 [38]	2011	South Africa: mobile testing units, Cape Metropolitan Region, Western Cape Province	HIV+ patients (192)	Aug 2008–Dec 2009	Phone follow-up of patients who tested positive at mobile testing units, with confirmation by record review
Tanzania 1 [39]	2009	Tanzania: VCT site and clinic in Kisesa Ward	HIV+ patients (349)	Mar 2005–Feb 2008	Evaluation of referral forms
Uganda 1 [40]	2009	Uganda: AIDS Support Clinic, Jinja	ART-eligible patients (2,483)	Sep 2004–Dec 2006^c^	Retrospective cohort
Uganda 2 [41]	2010	Uganda: Mulago Hospital, Kampala	HIV+ in-patients (208)	Mar 2004–Mar 2005^c^	Trial of offering HIV test during inpatient stay or referral to outpatient HIV test after discharge; limited to previously hospitalized patients
Uganda 3 [42]	2011	Uganda: Immune Suppression Syndrome (ISS) Clinic, Mbarara	ART-eligible patients (2,639)	Oct 2007–Jan 2011	Retrospective cohort

a Used data for 2006 only because data provided for earlier years were incomplete.

b Samples in SA 2, SA 3, and SA 11 may overlap.

c Follow-up may have continued beyond this date; source ambiguous.

CSW, commercial sex worker; IDU, intravenous drug user; PMTCT, prevention of mother-to-child transmission; TB, tuberculosis; VCT, voluntary counseling and testing.

### Stage 1: Testing to Staging

**Figure 3:**
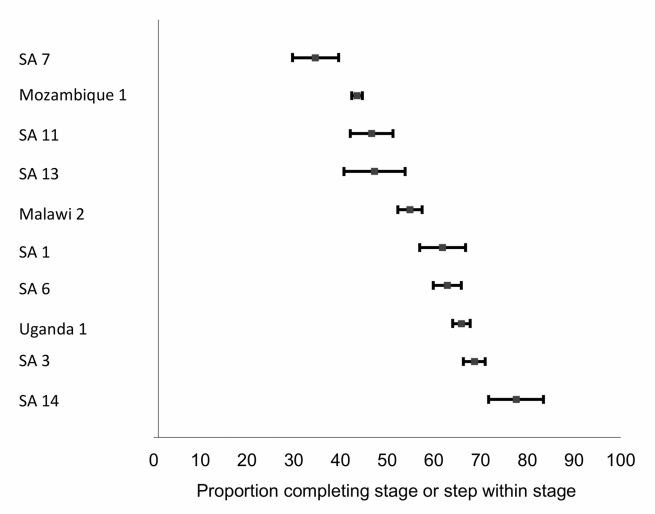
Forest plot of the ten studies reporting on the proportion of patients completing Stage 1 or steps within Stage 1. Bars indicate 95% confidence intervals. Studies shown in the plot report to differing end points; refer to Table 2 for details.

Ten studies reported rates of staging after a positive HIV test (Table 2 and Figure 3). Time intervals for evaluating results varied widely, from 1 wk to 6 mo. In general, between one-third and two-thirds of patients testing positive for HIV provided samples for CD4 counts and/or returned for results within 2–3 mo of the HIV test. For all the studies in Table 2, the median proportion of patients completing one or both of the steps in Stage 1 was 59% (range 35%–88%).

**Table 2:** Reported rates of retention or linkage in Stage 1 (HIV testing to staging).

Study Code	Outcome Assessed	*N*	Number Achieving Outcome	Percent (95% CI) Achieving Outcome	Comments
**Provided sample for CD4 count**					
SA 1	≤6 mo of HIV test	375	232	62% (57%–67%)	Source does not specify whether patients returned for results
SA 6	≤6 mo of HIV test	988	621	63% (60%–66%)	Source states that authors do not know whether patients returned for results; mean for those providing sample in >6 mo = 490 d
	>6 mo of HIV test	988	112	11% (9%–13%)	
	Never	988	255	26% (23%–29%)	
**Returned for CD4 count results after providing sample**					
Malawi 2	≤1 mo of registering for care	1,428	784	55% (52%–57%)	
SA 7	≤12 wk of HIV test	352	122	35% (30%–40%)	
SA 13	≤1 wk of providing sample	224	106	47% (41%–54%)	
SA 14	Ever	192	149	78% (72%–84%)	No maximum time limit indicated
Uganda 1	Ever	2,483	2,182	88% (87%–89%)	All patients enrolled in study were ART-eligible at time of providing CD4 count sample; no maximum time limit indicated
	Of above total, returned ≤21 d	2,483	1,637	66% (64%–68%)	
**Provided sample and returned for CD4 count results**					
Mozambique 1	≤60 d of HIV test	6,999	3,046	44% (42%–45%)	
	Of above total, enrolled in care ≤30 d of HIV test	7,005	3,950	56% (55%–58%)	
	Of above total, returned for CD4 results ≤30 d of enrollment	3,950	3,046	77% (76%–78%)	
SA 3	≤90 d of HIV test	1,474	1,012	69% (66%–71%)	Source is ambiguous but appears to refer to receipt of CD4 results, rather than solely provision of sample
SA 11	Ever	454	212	47% (42%–51%)	No maximum time limit is indicated for returning for results
	Of above total, provided sample for CD4 testing ≤8 wk of HIV test	454	248	55% (50%–59%)	
	Of above total, ever returned for results	248	212	85% (81%–89%)	No maximum time limit is indicated for returning for results

### Stage 2: Staging to ART Eligibility

**Figure 4:**
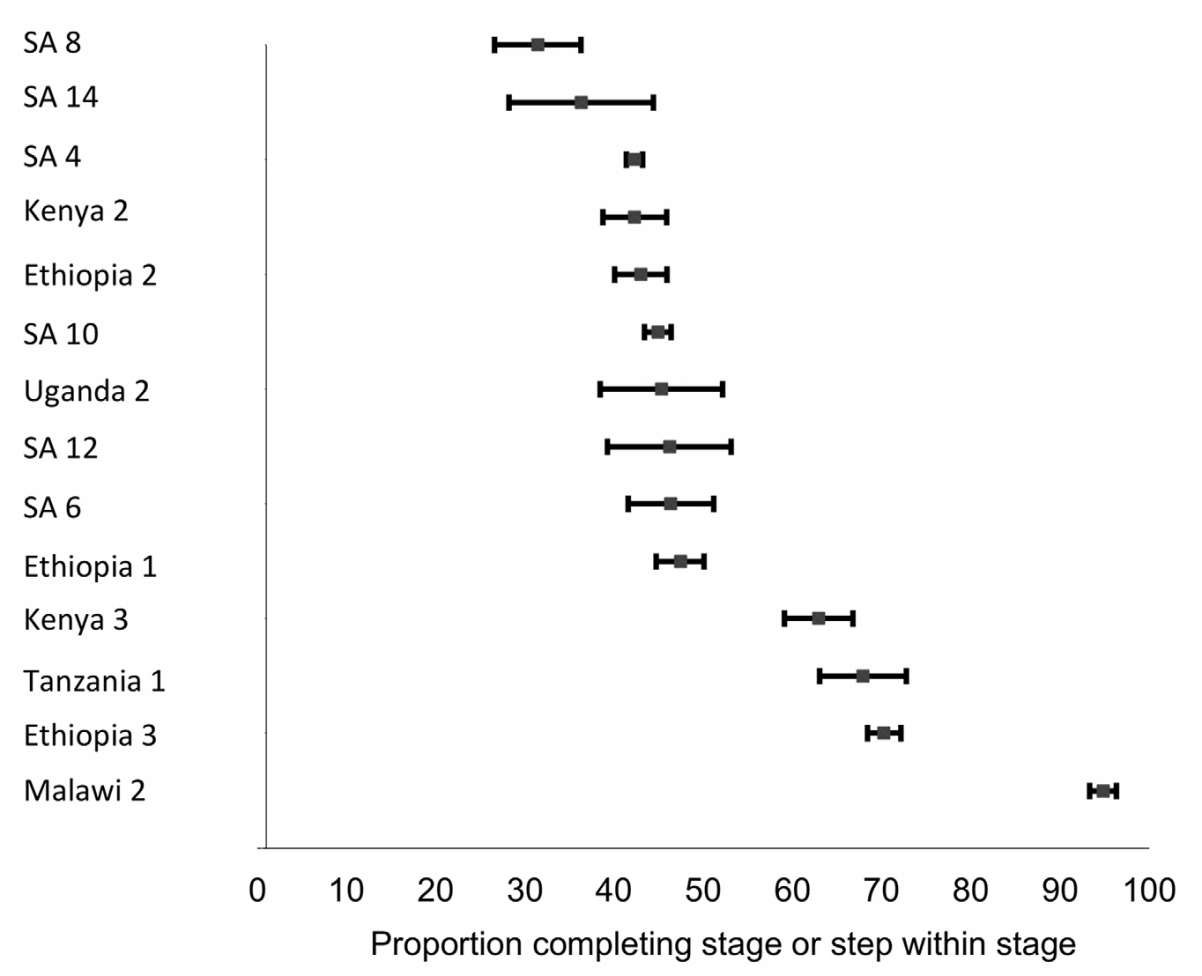
Forest plot of the 14 studies reporting on the proportion of patients completing Stage 2 or steps within Stage 2. Bars indicate 95% confidence intervals. Studies shown in the plot report to differing end points; refer to Table 3 for details.

Fourteen studies reporting on retention in pre-ART care between staging and ART eligibility (Stage 2) are shown in Table 3 and Figure 4. The upper rows of Table 3, which report on enrollment in pre-ART care after a positive HIV test, clearly overlap with some of the studies classified as Stage 1 and presented in Table 2, but we placed them in Stage 2 because they focus on pre-ART care rather than staging. Similarly, many of the studies in the lower rows of Table 3, which report on retention in pre-ART care after enrollment, use ART initiation as an end point, overlapping with Stage 3.

**Table 3 pmed-1001056-t003:** Reported rates of retention or linkage in Stage 2 (staging to ART eligibility).

Study Code	Outcome Assessed	*N*	Number Achieving Outcome	Percent (95% CI) Achieving Outcome	Comments
**HIV test to enrollment in care**					
Ethiopia 1	“Immediate” linkage to HIV care after HIV test	1,314	623	47% (45%–50%)	“Linked to care” and “immediately” not defined in report
Ethiopia 2	Visited referral site (HIV clinic) after HIV test	1,102	474	43% (40%–46%)	Of 474 visiting referral site, 84% visited ≤8 wk of HIV test
Kenya 2	Self-reported attendance at HIV care services 2–4 mo after HIV test	737	312	42% (39%–46%)	
SA 8	Attended first pre-ART medical appointment ≤1 y of staging	356	112	31% (27%–36%)	
SA 12	Visited referral site (HIV clinic) after HIV test	199	92	46% (39%–53%)	Self-reported data; time allowed to reach end point not stated
SA 14	Self-reported access of HIV care	135	49	36% (28%–44%)	Of those not linked, 1% died and 41% not reached by phone. Self-reported data confirmed by record review. Time limit for accessing care not clear
Tanzania 1	Registered at HIV clinic ≤6 mo of referral from testing	349	237	68% (63%–73%)	
Uganda 2	Self-reported attendance at HIV clinic ≤6 mo of HIV test	203	92	45% (39%–52%)	Self-reported data; denominator includes 55 patients who died ≤3 mo of HIV test
**Retention in pre-ART care after enrollment**					
Ethiopia 3	Percent initiating care or still in care at date of data censoring (follow-up duration unknown)	2,191	1,540	70% (68%–72%)	Of 651 not retained, 102 died and 549 lost to follow-up; proportion retained includes 34 who transferred out of program
Kenya 3	<30 d late for most recent pre-ART appointment or drug pickup 12 mo after enrollment in pre-ART care	610	384	63% (59%–67%)	Data are for period before distribution of cotrimoxazole to pre-ART patients
Malawi 2	Percent initiating care or still in care at date of data censoring (7 mo of follow-up)	852	808	95% (93%–96%)	45% of original sample did not return for CD4 count results and thus did not reach Stage 2; see Table 2
SA 10	Repeat CD4 count ≤13 mo of first CD4 count	4,223	1,896	45% (43%–46%)	
SA 4	Percent initiating care or still in care at date of data censoring (up to 3.5 y of follow-up)	11,039	4,672	42% (41%–43%)	Of 6,367 not retained, 1,337 died and 5,030 lost to follow-up
SA 6	Repeat CD4 count by date of data censoring (up to 5 y of follow-up)		191	46% (41%–50%)	

The first eight studies in Table 3 reported the proportion of patients who enrolled in an HIV care program after testing. Time intervals allowed for completing this step varied from “immediately” after the HIV test to a year after staging, and “enrollment in care” was itself not consistently defined. Across the studies, the median proportion of patients enrolling in care after a positive HIV test was 44% (range 31%–68%).

The last six studies in Table 3 reported on retention in ART care after enrollment. Four provided results up to the date of data censoring, rather than up to a clinically meaningful end point within the stage or to a consistent duration of follow-up of all patients in the cohort. In most of these cases, the outcome assessed was the proportion of patients who either initiated ART or remained in pre-ART care at the censoring date. In these studies, a median of 55% (range 42%–95%) of patients reached the study end point (repeat CD4 count, ART initiation, 12 months of follow-up, or data censoring), while the rest died or were lost to follow-up before the end point.

### Stage 3: ART Eligibility to ART Initiation

**Figure 5:**
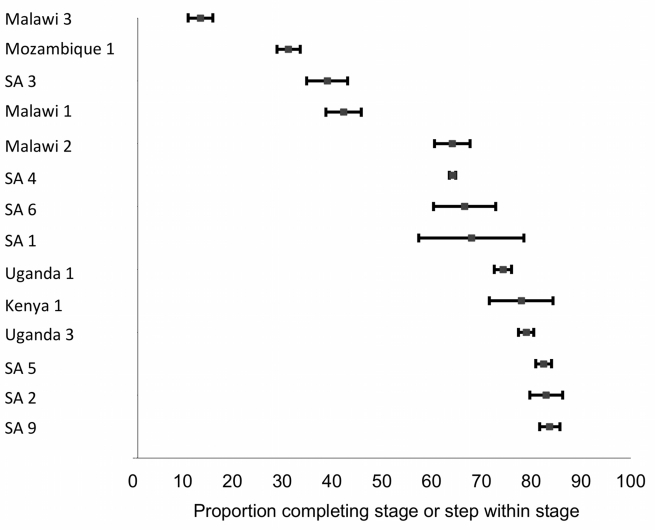
Forest plot of the 14 studies reporting on the proportion of patients completing Stage 3 or steps within Stage 3. Bars indicate 95% confidence intervals. Studies shown in the plot report to differing end points; refer to Table 4 for details.

The 14 studies reporting on Stage 3 are summarized in Table 4 and illustrated in Figure 5. Stage 3 has the most consistent and precise start and end points: from a clearly defined threshold, treatment eligibility, to a definite event, ART initiation. Across all the studies in Table 4, a median of 68% (range 14%–84%) of patients eligible for ART actually initiated treatment within the study periods of observation. As with Stage 1 and Stage 2, the time intervals allowed for the completion of Stage 3 varied widely and were in some cases unclear.

**Table 4:** Reported rates of retention or linkage in Stage 3 (ART eligibility to ART initiation).

Study Code	Time Interval Allowed for ART Initiation	*N*	Number Initiating ART	Percent (95% CI) Initiating ART	Comments
Kenya 1	By date of data censoring (no time limit indicated)	159	124	78% (71%–84%)	
Malawi 1	Not stated	742	314	42% (39%–46%)	Limited to ART-eligible pregnant women
Malawi 2	By date of data censoring	681	437	64% (61%–68%)	
Malawi 3	≥8 wk after starting TB treatment (no time limit indicated)	742	101	14% (11%–16%)	Limited to TB patients; ART eligibility based on TB diagnosis
Mozambique 1	≤90 d of eligibility	1,506	471	31% (29%–34%)	
SA 1	≤6 mo of eligibility	75	51	68% (57%–78%)	
SA 2	≤3 mo of last required pre-ART visit	501	416	83% (80%–86%)	Includes eight patients still preparing to start ART; of those not initiating ART, 82 lost to follow-up and three died
SA 3	By date of data censoring (median follow-up of 12 mo)	538	210	39% (35%–43%)	
SA 4	By date of data censoring (up to 3.5 y of follow-up)	22,083	14,179	64% (64%–65%)	Includes 1,216 patients still in pre-ART care at date of data censoring
SA 5		2,131	1,758	82% (81%–84%)	Weighted average of results for pregnant and non-pregnant patients; time interval not stated
SA 6	≤6 mo of HIV test if ART eligibility confirmed ≤6 mo of HIV test	219	146	67% (60%–73%)	
SA 9	By date of data censoring (3 y of follow-up)	1,235	1,034	84% (82%–86%)	
Uganda 1		2,483	1,846	74% (73%–76%)	Time interval not stated
Uganda 3	≤1 y of enrollment in care if ART-eligible at enrollment	2,639	2,085	79% (77%–81%)	

TB, tuberculosis.

### Multi-Stage Follow-Up

Eight studies contained data pertaining to more than one stage between testing and treatment initiation. These studies are listed multiple times in Tables 2–4 above, but the patient samples assessed at each stage did not remain consistent between stages in all of the studies. In Table 5, we provide multi-stage results for the relevant studies. Even the most comprehensive studies, such as Malawi 2 and SA 6, did not report outcomes to the end of a stage for all patients enrolled. Four of the seven studies that started with Stage 1 followed through to Stage 3 only those patients who were already eligible for ART in Stage 1—no further follow-up was reported of patients who were not yet eligible for ART and should have progressed to Stage 2.

**Table 5:** Proportions of patients retained between stages of pre-ART care.

Study Code	Stages and Start and End Points	Study Outcome	*N*	Number Achieving Outcome	Percent Achieving Outcome	Comments
Malawi 2	Stages 1–3. HIV testing to staging, retention in pre-ART care, and ART eligibility to ART initiation	Proportions of patients in WHO stage 1 or 2 and CD4 >250 cells/mm^3^ at enrollment who enrolled in HIV care, provided sample for CD4 count, and initiated ART by date of data censoring	1,633	808	49%	95% of losses to follow-up occurred in Stage 1; does not report stage completion for patients still in pre-ART care at data censoring
SA 6	Stages 1–3. HIV testing to staging, retention in pre-ART care, and ART eligibility to ART initiation	Proportion who initiated ART or had a repeat CD4 count by date of data censoring	988	330	33%	Does not report stage completion for patients not eligible for ART upon receipt of first CD4 count results
SA 4	Stages 2 and 3. Staging to ART initiation or data censoring	Proportion of those enrolled in program and with CD4 count reported who initiated ART or remained in care at date of data censoring	33,122	18,851	57%	Does not report stage completion for patients not eligible for ART upon receipt of first CD4 count results
SA 14	Stages 1 and 2. HIV testing to staging, and staging to enrollment in care	Proportion who returned for CD4 count results	192	149	77%	Does not report time limit for completing steps
		Proportion of those who returned for CD4 count results who reported accessing HIV care	135	49	36%	
Mozambique 1	Stages 1 and 3. HIV testing to staging, and ART eligibility to ART initiation	Proportion who returned for CD4 count results ≤60 d of HIV test	6,999	3,046	44%	Does not report outcomes for patients not eligible for ART upon receipt of CD4 count results
		Proportion of those ART-eligible at first CD4 count who initiated ART ≤90 d of CD4 count	1,506	417	31%	
SA 1	Stages 1 and 3. HIV testing to staging, and ART eligibility to ART initiation	Proportion who had C4 count ≤6 mo	375	233	62%	Does not report outcomes for patients not eligible for ART upon receipt of CD4 count results
		Proportion of those ART-eligible at first CD4 count who initiated ART ≤6 mo of HIV test	75	51	68%	
SA 3	Stages 1 and 3. HIV testing to staging, and ART eligibility to ART initiation	Proportion who returned for CD4 count results ≤90 d of HIV test	1,474	1,012	69%	Does not report outcomes for patients not eligible for ART upon receipt of CD4 count results
		Proportion of those ART-eligible at first CD4 count who initiated ART ≤12 mo of CD4 count	538	210	39%	
Uganda 1	Stages 1 and 3. HIV testing to staging, and ART eligibility to ART initiation	Of those who provided samples for CD4 count and were ART-eligible, proportion initiating ART vwithin an unspecified time period (<1 y)	2,483	1,846	74%	Excluded patients not yet ART-eligible at time of first CD4 count

## Discussion

During the early years of HIV/AIDS treatment scale up in sub-Saharan Africa, attention was focused on initiating eligible patients on ART and, more recently, on long-term retention in care of those patients on treatment. Growing awareness of the negative consequences of late presentation for treatment, combined with new enthusiasm for test-and-treat strategies, is now leading to renewed interest in the pre-ART period, which is after HIV diagnosis but before treatment.

**Figure 6:**
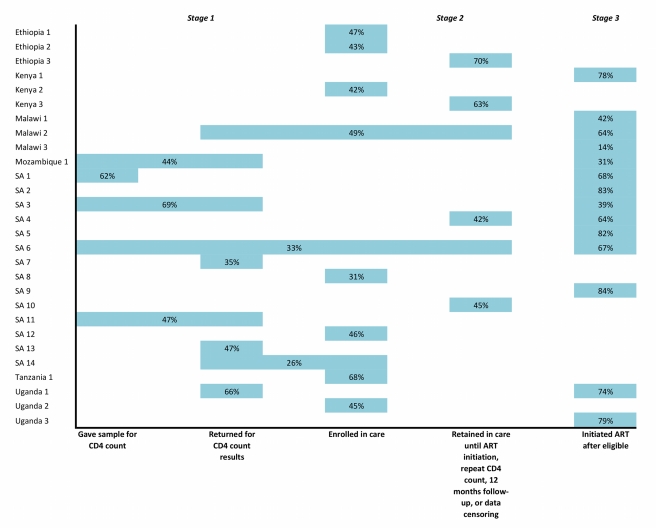
Summary of proportions of patients completing steps within each stage of pre-ART care in the studies reviewed.

Our analysis of 24 studies documenting rates of retention of patients from testing positive for HIV infection to initiating ART suggests that patient management during this period poses serious challenges. Most studies reported a substantial reduction in patient numbers at every step of the process. This reduction in patient numbers is clearly illustrated in Figure 6, which summarizes findings from all the reports. Studies are few, however, and offering a definitive answer to our core question—what proportion of patients who test positive for HIV are staged, enroll and remain in pre-ART care until ART eligibility, and initiate ART—is not possible with the data available. Only a handful of countries are represented, and most by no more than one or two studies. No study provides all the information needed to answer this question, even for a single setting, and combining results from multiple studies appears ill-advised. To examine the implications of doing this, we multiplied the median proportions of patients achieving the study end point in each stage (Stage 1, 59%; Stage 2, 46%; Stage 3, 68%), and found that the information available suggests that only about 18% of patients who are not yet eligible for ART when they are diagnosed with HIV remain continuously in care until ART eligibility. When we instead multiplied all combinations of estimates from each of the three stages, we estimated a median completion of all three stages of 17%, with an interval from the 10th to the 90th percentile of 7%–32%.

If we make one optimistic assumption, we can use the data in the most complete study in our review—SA 6, which tracked patients from provision of a sample for a CD4 count to either ART initiation or a repeat CD4 count—to answer the question for one setting. In SA 6, 988 patients were enrolled after testing positive for HIV. By the end of the study, 141 had initiated ART, and 189 had returned for at least one repeat CD4 count. If we optimistically assume all 189 in the latter group remained in pre-ART care until ART initiation, then the overall retention rate for this population was 33%, better than what we estimated by multiplying the medians but still very low. While it is difficult to believe that only a sixth to a third of patients remain continuously in care, the evidence does not allow us to make a more definitive estimate.

There appear to be several main reasons for the poor performance of pre-ART care in retaining patients. Most patients during this stage are asymptomatic and may not perceive themselves as requiring medical care. Since very little therapeutic care is offered during the pre-ART period, patients must take it on faith that making the effort to come to the clinic for monitoring is worth the costs of doing so. Current approaches to providing care often require multiple clinic visits, for example, to first provide a blood sample for a CD4 count and then return a week later to receive the results. Choosing to “wait and see what happens” may well be a preferred strategy for patients who lack resources for transport, risk losing employment by taking time off work, or fear being recognized as a client of an HIV clinic. Other patients, those who already have very low CD4 counts at their first presentation for HIV care, do not complete Stage 3 because they die before doing so. A number of the papers we reviewed stratified results by CD4 count range and/or identified other factors associated with pre-ART attrition, and a review of these findings would be valuable.

In interpreting the results summarized above, it should also be kept in mind that there is far more mobility among HIV patients than had been anticipated [8]. Loss to follow-up at any one site may or may not indicate that a patient has dropped out of care permanently. Some patients may have returned to the same site after the data for the study were censored or the study's definition of loss to care reached. Many patients may have simply transferred, usually informally, from one site to another. Difficult as this problem is for managing ART patients, it is even worse during the pre-ART period, because patients are expected to visit the clinic less frequently, and more clinics are able to provide pre-ART services than are accredited to offer ART. For individual patients, dropping out of pre-ART care is less likely to represent a death sentence than is loss to follow-up after initiating treatment. Patients lost to pre-ART care mainly risk becoming late presenters to treatment, not dying. It is reasonable to assume that many, if not most, patients who drop out of pre-ART care will return to the health-care system at some later date, most likely once they become seriously ill. Without an effective health information system that allows patients to be tracked from site to site and over time, as they come and go from care, it is nearly impossible to assess the extent to which patient mobility mitigates the observed loss to care rates.

While pre-ART loss to care may not pose as immediate a mortality threat as loss of patients who already have clinical AIDS, it is still a major impediment to improving the outcomes of HIV care and treatment overall, is itself a contributor to the high mortality observed during the first year on ART, and wastes scarce health system resources. What can be done to begin to address this problem? We have heard of several operational solutions currently being evaluated, involving adjustments in referral procedures, improvements in the information provided to patients, reminders conveyed by text message or phone, or an increase in the number of steps that can be completed in a single visit. We have seen few rigorous evaluations of interventions, however. One exception, which is currently being evaluated in several settings, is the use of point-of-care CD4 count technology to reduce the number of visits to the clinic in Stage 1 [9]–[13]. Another promising strategy is to dispense prophylaxis for opportunistic infections, such as cotrimoxazole and isoniazid, more actively to pre-ART patients; a study in Kenya reported that retention of pre-ART patients 12 mo after enrollment improved from 63% to 84% after provision of cotrimoxazole was introduced [14].

### Research Priorities

A discussion of interventions is beyond the scope of this paper but would warrant further investigation. What we do wish to discuss are two issues that arise directly from this review. First, the review made painfully clear the need for standardization of terminology, definitions, time intervals, and end points that should be reported for the pre-ART period. The three-stage structure presented here may provide a framework for classifying results, but it is no more than a starting point. We have three recommendations for how researchers might begin to address this issue. First, proposals for clearly defined outcomes within each stage, and standard terminology to describe those outcomes and to label the phenomenon of pre-ART loss to care overall, would be helpful. Suggestions from researchers involved in work in this area, and thus familiar with data availability and limitations, would be welcome. Second, more effort should be made to report quantitative data comprehensively. We were forced to exclude from our review one paper and several conference abstracts that indicated that the authors likely had the data required to make quantitative estimates of retention in pre-ART care but did not report them or reported them incompletely. Having a standard set of indicators and outcomes, as suggested above, would also help to solve this second problem. And third, using data censoring as an end point should be avoided when possible, in favor of a clinically meaningful end point or a fixed duration of follow-up.

The second issue highlighted by this review is the absence of health information systems that allow patients to be tracked between service delivery points. We did not find a single study that was able to follow a cohort of HIV-positive adults all the way from testing to treatment initiation if they were not already eligible for ART when diagnosed. While in retrospect this points to a failure of the research community to establish prospective cohorts several years ago, it also reflects the sheer difficulty posed by such research. In most settings we are familiar with, it is virtually impossible to determine retrospectively what happens to patients after testing positive for HIV, as there is no tracking system in place to indicate whether they have sought further care or not. In our experience, even where sophisticated electronic record systems are in use for managing ART patients, they are rarely kept up to date or complete for those who have not initiated ART.

A starting point for understanding the nature and scope of the problem of pre-ART loss to care might thus be to implement effective patient tracking systems in selected geographic catchment areas that will generate accurate information on attrition between and within stages and help researchers assess the role of patient mobility in offsetting observed attrition, identify characteristics of patients most likely to be lost, and explore the extent to which attrition from pre-ART care is temporary—i.e., delay in action by patients who will later return to care, albeit sicker—or represents permanent loss from the health-care system, which will likely ultimately lead to death. Even doing this on a relatively small scale will be challenging, as it has been for ART patients [15], but it is a vital intervention for improving pre-ART care.

### Limitations and a Call for Data

The heterogeneity of the literature identified, and the sheer scarcity of studies found for most sub-Saharan countries, led to a number of review limitations that are important to bear in mind in interpreting our findings. Most of these limitations have been alluded to already but warrant reiteration here. First, the quality and heterogeneity of the studies prevented meaningful synthesis of the results, which should therefore be regarded as suggestive rather than conclusive. The lack of standard definitions among reports, or even clear definitions of outcome measures within some (but not all) of the reports, combined with inconsistent or unreported durations of follow-up, stymied aggregate analysis. This limitation should be kept in mind in interpreting the forest plots and the summary figure (Figure 6) in particular. Second, double-counting likely affects some of the studies. Patients who are lost from one stage of care can return to care later and either successfully complete the stage or be lost again. Single-stage studies can tell us whether patients remain continuously in care until the end of the stage but should not be combined with studies of other stages, as demonstrated by our multiplying of median estimates above. Third, there is likely important heterogeneity among study populations that could not be discerned from most reports. For example, patients who enroll in pre-ART care (Stage 2) with low CD4 counts, close to the ART eligibility threshold, have less time at risk of being lost from care than those who enroll earlier, with higher CD4 counts, but few studies reported this information. Fourth, half of the studies eligible for inclusion in our review came from just one country, South Africa, and only six other countries are represented by the rest of the studies. This may diminish the generalizability of the findings to the sub-Saharan region as a whole. Fifth, eight of the 28 studies included were in abstract form only and were thus not subjected to peer review. Finally, publication bias may have affected our summary estimates. Only a few HIV clinics in sub-Saharan Africa have published information about pre-ART loss to care, and most of these sites collaborate with nongovernmental organizations, universities, or other external partners. If sites that have the ability and resources to report on such data have either lower or higher than average retention rates, our summary estimates will be biased.

Needless to say, new health information systems or studies launched now—the best solution to the problems described above—will require several years to accumulate the duration of follow-up needed. We therefore conclude with a call to HIV/AIDS service delivery organizations in the field. We think it likely that some programs have captured the data needed to analyze pre-ART loss to care through all three stages. We speculate that in some geographic areas, a single organization is the sole provider of every step of HIV care and treatment delivery. If that organization has also assigned a unique patient identification number to all those served, beginning with HIV testing, then an adequate data set may exist. We hope that this paper will inspire those who may have such data to try to answer the questions raised here, and that we will soon begin to see the results of this effort in the literature.

## Supporting Information

Text S1 **Search protocol.**
(DOC)

Text S2 **PRISMA checklist.**
(DOC)

## Abbreviations

ART: antiretroviral therapy

## References

[1] World Health Organization, Joint United Nations Programme on HIV/AIDS, United Nations Children's Fund (2010). Towards universal access: scaling up priority HIV/AIDS interventions in the health sector; progress report 2010.

[2] Fox MP, Rosen S (2010). Patient retention in antiretroviral therapy programs up to three years on treatment in sub-Saharan Africa, 2007–2009: systematic review.. Trop Med Int Health.

[3] Brinkhof MW, Dabis F, Myer L, Bangsberg DR, Boulle A (2008). Early loss of HIV-infected patients on potent antiretroviral therapy programmes in lower-income countries.. Bull World Health Organ.

[4] Leisegang R, Cleary S, Hislop M, Davidse A, Regensberg L (2009). Early and late direct costs in a Southern African antiretroviral treatment programme: a retrospective cohort analysis.. PLoS Med.

[5] Alcorn K (2010). South Africa to launch mass HIV testing drive in April, to test 15 million in one year.. http://www.aidsmap.com/South-Africa-to-launch-mass-HIV-testing-drive-in-April-to-test-15-million-in-one-year/page/1438260/.

[6] Cornell M, Grimsrud A, Fairall L, Fox MP, van Cutsem G (2010). Temporal changes in programme outcomes among adult patients initiating antiretroviral therapy across South Africa, 2002–2007.. AIDS.

[7] Keiser O, Anastos K, Schechter M, Balestre E, Myer L (2008). Antiretroviral therapy in resource-limited settings 1996 to 2006: patient characteristics, treatment regimens and monitoring in sub-Saharan Africa, Asia and Latin America.. Trop Med Int Health.

[8] Geng EH, Nash D, Kambugu A, Zhang Y, Braitstein P (2010). Retention in care among HIV-infected patients in resource-limited settings: emerging insights and new directions.. Curr HIV/AIDS Rep.

[9] Jani I, Sitoe N, Alfai E, Chongo P, Lehe J (2010). Point-of-care CD4 improves patient retention and time-to-initiation of ART in Mozambique [abstract]..

[10] Faal M, Naidoo N, Makgamatha L, Venter F, Osih R (2010). Effect of an immediate CD4 result during VCT on patient retention in ART [abstract]..

[11] Mtapuri-Zinyowera S, Chideme M, Mangwanya D, Mugurungi O, Gudukeya S (2010). Evaluation of the PIMA point-of-care CD4 analyzer in VCT clinics in Zimbabwe.. J Acquir Immune Defic Syndr.

[12] Larson BA, Ndibongo B, Brennan A, Bistline K, Xulu T (2011). Point-of-care CD4 testing after HIV diagnosis to reduce losses to initiation of antiretroviral therapy: an evaluation of a pilot program at the Themba Lethu Clinic, Johannesburg, South Africa [abstract]..

[13] Larson BA, Bistline K, Ndibongo B, Xulu T, Brennan A (2011). Rapid point-of-care CD4 testing at mobile HIV testing sites to increase linkage to care: an evaluation of a pilot program in South Africa [abstract]..

[14] Kohler P, Chung M, Benki-Nugent S, McGrath C, Attwa M (2011). Free CTX substantially improves retention among ART-ineligible clients in a Kenyan HIV treatment program [abstract]..

[15] Fraser HS, Allen C, Bailey C, Douglas G, Shin S (2007). Information systems for patient follow-up and chronic management of HIV and tuberculosis: a life-saving technology in resource-poor areas.. J Med Internet Res.

[16] Assefa Y, Van D, Mariam DH, Kloos H (2010). Toward universal access to HIV counseling and testing and antiretroviral treatment in Ethiopia: looking beyond HIV testing and ART initiation.. AIDS Patient Care STDS.

[17] Berhanu AL (2009). Confirmed referral for pre-ART and ART services: best practices from USAID/PSP-Ethiopia mobile HIV counseling and testing [abstract]..

[18] Mulissa Z, Jerene D, Lindtjørn B (2010). Patients present earlier and survival has improved, but pre-ART attrition is high in a six-year HIV cohort data from Ethiopia.. PLoS ONE.

[19] Karcher H, Omondi A, Odera J, Kunz A, Harms G (2007). Risk factors for treatment denial and loss to follow-up in an antiretroviral treatment cohort in Kenya.. Trop Med Int Health.

[20] Amolloh M, Medley A, Owuor P, Audi B, Sewe M (2011). Factors associated with early uptake of HIV care and treatment services after testing HIV+ during home-based testing and counseling in rural Western Kenya [abstract]..

[21] Gareta D, Tweya H, Weigel R, Phiri S, Chiwoko J (2010). Linking HIV-infected pregnant women to antiretroviral therapy: experience from Lilongwe, Malawi [abstract]..

[22] Tayler-Smith K, Zachariah R, Massaquoi M, Manzi M, Pasulani O (2010). Unacceptable attrition among WHO stages 1 and 2 patients in a hospital-based setting in rural Malawi: can we retain such patients within the general health system?. Trans R Soc Trop Med Hyg.

[23] Zachariah R, Harries AD, Manzi M, Gomani P, Teck R (2006). Acceptance of anti-retroviral therapy among patients infected with HIV and tuberculosis in rural Malawi is low and associated with cost of transport.. PLoS ONE.

[24] Micek M, Gimbel-Sherr K, Baptista AJ, Matediana E, Montoya P (2009). Loss to follow-up of adults in public HIV care systems in central Mozambique: identifying obstacles to treatment.. J Acquir Immune Defic Syndr.

[25] April MD, Walensky RP, Chang Y, Pitt J, Freedberg KA (2009). Testing rates and outcomes in a South African community, 2001–2006: implications for expanded screening policies.. J Acquir Immune Defic Syndr.

[26] Bassett IV, Wang B, Chetty S, Mazibuko M, Bearnot B (2009). Loss to care and death before antiretroviral therapy in Durban, South Africa.. J Acquir Immune Defic Syndr.

[27] Bassett IV, Regan S, Chetty S, Giddy J, Uhler LM (2010). Who starts antiretroviral therapy in Durban, South Africa?…not everyone who should.. AIDS.

[28] Ingle SM, May M, Uebel K, Timmerman V, Kotze E (2010). Outcomes in patients waiting for antiretroviral treatment in the Free State Province, South Africa: prospective linkage study.. AIDS.

[29] Kaplan R, Orrell C, Zwane E, Bekker LG, Wood R (2008). Loss to follow-up and mortality among pregnant women referred to a community clinic for antiretroviral treatment.. AIDS.

[30] Kranzer K, Zeinecker J, Ginsberg P, Orrell C, Kalawe NN (2010). Linkage to HIV care and antiretroviral therapy in Cape Town, South Africa.. PLoS ONE.

[31] Larson BA, Brennan A, McNamara L, Long L, Rosen S (2010). Lost opportunities to complete CD4+ lymphocyte testing among patients who tested positive for HIV in South Africa.. Bull World Health Organ.

[32] Larson BA, Brennan A, McNamara L, Long L, Rosen S (2010). Early loss to follow up after enrolment in pre-ART care at a large public clinic in Johannesburg, South Africa.. Trop Med Int Health.

[33] Lawn SD, Myer L, Harling G, Orrell C, Bekker LG (2006). Determinants of mortality and nondeath losses from an antiretroviral treatment service in South Africa: implications for program evaluation.. Clin Infect Dis.

[34] Lessells RJ, Mutevedzi PC, Cooke GS, Newell ML (2011). Retention in HIV care for individuals not yet eligible for antiretroviral therapy: rural KwaZulu-Natal, South Africa.. J Acquir Immune Defic Syndr.

[35] Losina E, Bassett IV, Giddy J, Chetty S, Regan S (2010). The “ART” of linkage: pre-treatment loss to care after HIV diagnosis at two PEPFAR sites in Durban, South Africa.. PLoS ONE.

[36] Luseno W, Wechsberg W, Middlesteadt-Ellerson R, Gumula W (2008). Linkages and barriers to care for high-risk South African women testing positive for HIV [abstract]..

[37] Naidoo NP, Faal M, Venter WDF, Osih R (2010). Patient retention-reasons why patients do or do not come back for care after HIV testing [abstract]..

[38] Govindasamy D, van Schaik N, Kranzer K, Mpali M, Thuebus E (2011). Linkage to HIV care from a mobile testing unit in South Africa by different CD4 count strata [abstract]..

[39] Nsigaye R, Wringe A, Roura M, Kalluvya S, Urassa M (2009). From HIV diagnosis to treatment: evaluation of a referral system to promote and monitor access to antiretroviral therapy in rural Tanzania.. J Int AIDS Soc.

[40] Amuron B, Namara G, Birungi J, Nabiryo C, Levin J (2009). Mortality and loss-to-follow-up during the pre-treatment period in an antiretroviral therapy programme under normal health service conditions in Uganda.. BMC Public Health.

[41] Wanyenze RK, Hahn J, Liechty C, Ragland K, Ronald A (2011). Linkage to HIV care and survival following inpatient HIV counseling and testing.. AIDS Behav.

[42] Geng E, Muyindike W, Glidden D, Bwana M, Yiannoutsos CT (2011). Failure to initiate ART, loss to follow-up and mortality among HIV-infected patients during the pre-ART period in Uganda: Understanding engagement in care in resource-limited settings [abstract]..

