# Supplementary material for: Retention in HIV Care between Testing and Treatment in Sub-Saharan Africa: A Systematic Review
Source: PLoS Med. 2011 Jul 19;8(7):e1001056. doi: 10.1371/journal.pmed.1001056 (PMC3139665; doi:10.1371/journal.pmed.1001056)
Supplement: Text S1 — Search protocol. (DOC) [file pmed.1001056.s001.doc]

**Search protocol for systematic review of linkage to care in sub-Saharan Africa**

December 17, 2010

Authors

Sydney Rosen

Matt Fox

Background/justification

Very little is known about losses to follow up of patients between HIV testing and ART initiation in Africa, but preliminary evidence suggests that retention is low. Several recent papers have evaluated rates of linkage at specific points between testing and ART in various African countries. There is little consistency int their approach or definitions, however, making interpretation difficult. A review and compilation of these estimates would be of value to policy makers and program managers and for targeting interventions.

Review questions

1. For patients testing HIV-positive, what are the rates of linkage from testing to staging, staging to ART eligibility, and ART eligibility to ART initiation?
2. What is the nature of the published research on this issue, in terms of quantity, clarity, completeness, geographic representativeness, and quality?

Search strategy

*Electronic databases*

| **Database** | **Search terms** |
| --- | --- |
| Pubmed to January 5, 2011 | - Africa + HIV+ retention - Africa + HIV + linkage - Africa + HIV + pre-ART |
| ISI Web of Knowledge to January 5, 2011 | - Africa + HIV+ retention - Africa + HIV + linkage - Africa + HIV + pre-ART |
| African Indicus Medicus to April 1, 2011 | - HIV + treatment - HIV + linkage - HIV + retention - HIV + pre-ART - HIV + care |
| IAS abstract archives 2008-2010* | - Retention - Linkage |
| CROI abstract archives 1997-2011 | - Retention - Linkage - Pre-ART |
| Implementers’ Meeting abstract archives 2008-2009*^†^ | Scanned titles |
| IAPAC Adherence Conference 2010** | Scanned titles |

*Includes AIDS 2010, IAS 2009, and AIDS 2008. Little research was published on this topic prior to 2008, so IAS conference abstracts are searched only from 2008 to the present.

^†^No conference held in 2010.

**Previous years not available

*Manual searching*

Scan reference lists of articles identified through electronic database search

Scan tables of contents of all issues of African Journal of AIDS Research and Southern African Journal of HIV Medicine

Study selection criteria

Inclusion criteria

- Geographic range: sub-Saharan Africa
- Topic: retention of patients in care after HIV diagnosis and before (up to) ART initiation
- Design: cohort or clinic-level analysis with primary data, including program evaluations
- Setting: standard service delivery in any sector (public, nongovernmental, private) that serves the general population
- Population: adult patients (including sub-populations of pregnant women and TB patients)
- Minimum information included: must report rates of linkage between at least two defined points (intention to treat approach)
- Language of publication: no restrictions

Exclusion criteria:

- Geographic range: outside sub-Saharan Africa
- Topic: anything other than linkage between testing and treatment (retention in pre-ART care)
- Design: modeled estimates and non-representative samples, qualitative studies, as treated (rather than intention to treat) designs
- Setting: clinical trial (other than routine operational changes), including phase 3 trials, or other non-standard setting
- Population: pediatric patients or specific subsets of general population, ART for PMTCT only
- Minimum information included: cannot estimate proportions linked or retained between at least two defined points or reports as-treated only (does not report patients lost to care)

Study quality assessment

Because the review aims to assess public health practice, sources will not be excluded on the basis of quality provided that they meet all the inclusion criteria and none of the exclusion criteria for the review.

First author will do initial screen of sources and assess for inclusion. Others author will screen those found to meet the inclusion criteria. Disagreements will be resolved by consensus.

If more than one source reports information on the same cohort of patients or from the same clinics, include the one that contains the most complete or clearest information.

Data extraction strategy

The following fields will be extracted for each source reviewed:

- Source reference
- Source type (journal article, abstract)
- Country and location/site within country
- Sector and provider
- Facility level (hospital, clinic, other) and # facilities included
- Points of analysis (e.g. testing to staging, eligibility to initiation, etc.)
- Dates of data (enrollment/follow up)
- Population included and sample size
- Rates of retention/loss between points
- Definitions of lost categorization (e.g. number of weeks late)
- Other information to explain findings

Plan for synthesis of extracted data

- Describe studies
- Report rates of linkage or loss at each step between testing and ART initiation, with confidence intervals where possible
- Summarize by stage using medians and ranges
